# Therapeutic Assessment of Combination Therapy with a Neprilysin Inhibitor and Angiotensin Type 1 Receptor Antagonist on Angiotensin II–Induced Atherosclerosis, Abdominal Aortic Aneurysms, and Hypertension[Fn FN5]

**DOI:** 10.1124/jpet.121.000525

**Published:** 2021-06

**Authors:** Yasir AlSiraj, Sean E. Thatcher, Ching Ling Liang, Heba Ali, Mark Ensor, Lisa A. Cassis

**Affiliations:** Department of Pharmacology and Nutritional Sciences, University of Kentucky, Lexington, Kentucky

## Abstract

Combined neprilysin (NEP) inhibition (sacubitril) and angiotensin type 1 receptor (AT1R) antagonism (valsartan) is used in the treatment of congestive heart failure and is gaining interest for other angiotensin II (AngII)-related cardiovascular diseases. In addition to heart failure, AngII promotes hypertension, atherosclerosis, and abdominal aortic aneurysms (AAAs). Similarly, NEP substrates or products have broad effects on the cardiovascular system. In this study, we examined NEP inhibition (with sacubitril) and AT1R antagonism (with valsartan) alone or in combination on AngII-induced hypertension, atherosclerosis, or AAAs in male low-density lipoprotein receptor–deficient mice. Preliminary studies assessed drug delivery via osmotic minipumps for simultaneous release of sacubitril and/or valsartan with AngII over 28 days. Mice were infused with AngII (1000 ng/kg per minute) in the absence (vehicle) or presence of sacubitril (1, 6, or 9 mg/kg per day), valsartan (0.3, 0.5, 1, 6, or 20 mg/kg per day), or the combination thereof (1 and 0.3, or 9 or 0.5 mg/kg per day of sacubitril and valsartan, respectively). Plasma AngII and renin concentrations increased 4-fold at higher valsartan doses, indicative of removal of AngII negative feedback on renin. Sacubitril doubled plasma AngII concentrations at lower doses (1 mg/kg per day). Valsartan dose-dependently decreased systolic blood pressure, aortic atherosclerosis, and AAAs of AngII-infused mice, whereas sacubitril had no effect on atherosclerosis or AAAs but reduced blood pressure of AngII-infused mice. Combination therapy with sacubitril and valsartan did not provide additive benefits. These results suggest limited effects of combination therapy with NEP inhibition and AT1R antagonism against AngII-induced hypertension, atherosclerosis, or AAAs.

**SIGNIFICANCE STATEMENT:**

The combination of valsartan (angiotensin type 1 receptor antagonist) and sacubitril (neprilysin inhibitor) did not provide benefit above valsartan alone on AngII-induced hypertension, atherosclerosis, or abdominal aortic aneurysms in low-density lipoprotein receptor–deficient male mice. These results do not support this drug combination in therapy of these AngII-induced cardiovascular diseases.

## Introduction

In 2015, the US Food and Drug Administration approved Entresto (sacubitril/valsartan; [C96H120N12Na602]) for the treatment of heart failure. Entresto is a complex of sacubitril (C24H29N05), a neprilysin (NEP) inhibitor, and valsartan (C24H29N503), an angiotensin type 1 receptor (AT1R) antagonist (Gu et al., 2010). This combination was developed to treat heart failure because of the actions of sacubitril to inhibit NEP-mediated degradation of natriuretic peptides and the actions of valsartan to antagonize effects of angiotensin II (AngII) at AT1R (Lillyblad, 2015). NEP becomes an important pathway for degradation of beneficial vasodilating and sodium diuresis natriuretic peptides such as atrial or brain natriuretic peptide in patients with congestive heart failure (Koglin et al., 2001), and AngII is a powerful stimulant for cardiac hypertrophy and the release of aldosterone to increase sodium and water retention, which is detrimental for patients with heart failure (Benedict et al., 1994). Therefore, use of a combined NEP inhibitor with an AT1R antagonist through Entresto was developed and found to reduce the relative risk of cardiovascular death or first hospitalization by 20% in the Prospective Comparison of ARNI With ACEI to Determine Impact on Global Mortality and Morbidity in Heart Failure (PARADIGM-HF) trial (Packer et al., 2015). More recently, Prospective Study of Biomarkers, Symptom Improvement, and Ventricular Remodeling During Sacubitril/Valsartan Therapy for Heart Failure (PROVE-HR), a prospective, single-group, open-label study of Entresto in patients with heart failure with reduced ejection fraction demonstrated a 9.4% improvement in ventricular ejection fraction and favorable changes in left ventricular volumes and filling pressures (Januzzi et al., 2019). Although these results are clinically significant and support use of Entresto in patients with New York Heart Association (NYHA) class II–IV heart failure, mechanisms for the therapeutic benefit of NEP inhibition have remained elusive.

NEP has several substrates, including natriuretic peptides, adrenomedullin, endothelin, substance P, bradykinin, and AngII (Erdös and Skidgel, 1989; Bayes-Genis et al., 2016). Notably, NEP degrades AngII into angiotensin-(1–4) and angiotensin-(5–8), two inactive angiotensin peptide fragments (Schling and Schafer, 2002). This action of NEP seems paradoxical in reference to the effectiveness of NEP inhibition in heart failure, as levels of AngII may be expected to rise upon NEP inhibition. However, combination of NEP inhibition with an AT1R antagonist would be anticipated to minimize concerns over potential elevations in systemic AngII concentrations. Given the broad substrate specificity of NEP, it should be noted that several NEP substrates influence other cardiovascular responses. For example, similar to the vasoconstrictor AngII, NEP also degrades the powerful vasoconstrictor endothelin (Vijayaraghavan et al., 1990; Abassi et al., 1992; Ferro et al., 1998; Park et al., 2003); thus, NEP inhibition has been reported to result in elevated blood pressure in diabetic or hypertensive rats even when combined with blockade of the renin-angiotensin system (Roksnoer et al., 2015, 2016). Conversely, NEP also degrades vasodilators such as atrial natriuretic peptide, bradykinin, and substance P, and thus, NEP inhibition would be predicted to elevate levels of these vasodilator substances and decrease blood pressure. For these reasons, it is difficult to predict the overall impact of NEP inhibition, either alone or in combination with AT1R antagonism, on blood pressure, and the mode of elevating blood pressure would most likely influence antihypertensive efficacy of combined therapy with an NEP inhibitor and an AT1R antagonist.

We and others have demonstrated that infusion of AngII to hypercholesterolemic mouse models promotes the development of atherosclerosis and causes the formation of abdominal aortic aneurysms (AAAs) (Daugherty and Cassis, 1999; Daugherty et al., 2000). In addition to AngII, natriuretic peptides, especially brain natriuretic peptide, has been linked to experimental atherosclerosis in which NEP inhibition exerted favorable effects on lesion formation (Schirger et al., 2000). Similarly, brain natriuretic peptide and its N-terminal fragment have been suggested as biomarkers for postoperative cardiac events in patients undergoing surgery for AAAs (Feringa et al., 2006, 2007; Rajagopalan et al., 2008; Vetrugno et al., 2012; Bryce et al., 2013). Given that both of these NEP substrates, namely AngII and natriuretic peptides, have been linked to atherosclerosis and AAAs, in this study we hypothesized that combined therapy of NEP inhibition and AT1R antagonism would provide added benefit beyond either drug alone on AngII-induced hypertension, atherosclerosis, and AAA in hypercholesterolemic mice.

## Materials and Methods

### 

#### Mice and Drug Treatment.

Male low-density lipoprotein receptor–deficient (*Ldlr*^*−/−*^) mice (2 months of age; B6/129S7-Ldl^tm1Her^/J, stock 002207; Jackson Laboratory, Bar Harbor, MA) were randomized to groups such that baseline body weights of each group were not significantly different. Mice were fed a Western diet (TD.88137; Teklad, Dublin, VA) beginning 1 week prior to osmotic minipump implantation and through study endpoint. Osmotic minipumps (Alzet model 2001) containing either vehicle [USP-grade DMSO (Sigma, St. Louis, MO) in a 1:1 volume with propylene glycol (2FF0019; Spectrum, New Brunswick, NJ)], valsartan [0.3, 0.5, 1, 6, or 20 mg/kg per day (Wu et al., 2001; Yamamoto et al., 2009; Lei et al., 2012), PHR1313; Sigma], sacubitril [1, 6, or 9 mg/kg per day (Bai et al., 2015; Ishii et al., 2017), 21473; Cayman Chemical, Ann Arbor, MI)], or combinations thereof (sacubitril/valsartan, 1.0:0.3 or 9:0.5 mg/kg per day) were implanted subcutaneously in anesthetized mice for 7 days of pretreatment. At 1 week later, minipumps were removed from anesthetized mice and replaced with fresh minipumps (Alzet model 2004) containing pretreatment drug(s) with AngII (1000 ng/kg per minute; 4006473; Bachem, Torrance, CA) for 28 days of delivery (Daugherty and Cassis, 1999; Daugherty et al., 2000). At the study endpoint, mice were anesthetized (ketamine/xylazine, 100:10 mg/kg, i.p.) for exsanguination by cardiac puncture and tissue harvest. In addition, at the study endpoint, drug remaining in osmotic minipumps was routinely quantified to assure drug delivery. All studies were initiated at *n* = 10 mice per group. However, because studies were performed over an extended duration and all groups could not be performed simultaneously, vehicle groups infused with AngII only were included across all studies, and data from mice administered vehicle were then combined within data analysis and illustrated figures. All procedures were in accordance with the guidelines approved by the University of Kentucky Institutional Animal Care and Use Committee. We used male mice for these studies because female mice do not readily develop AAAs in response to AngII infusions (Henriques et al., 2004). Moreover, we used hypercholesterolemic-susceptible *Ldlr*^*−/−*^ mice as this genetic background, which, when fed a Western diet, has been demonstrated to exhibit susceptibility to each of the primary-endpoint AngII-induced responses (Daugherty and Cassis, 1999; Lu et al., 2016; Liu et al., 2020). Unless otherwise stated, we included data from all mice that completed the 28-day infusion protocol for endpoint measures (serum components, ultrasound aortic lumen diameter measurements, en face atherosclerosis quantification).

#### Assessment of Drug Release from Osmotic Minipumps.

We assessed release of sacubitril (9 mg/kg per day), valsartan (0.5 mg/kg per day), and AngII (1000 ng/kg per minute) from osmotic minipumps (model 2004; all three drugs placed within *n* = 3 osmotic minipumps). Minipumps were placed in sterile phosphate-buffered saline (2 ml) at 37°C. Fresh buffer was placed with osmotic minipumps on days 7, 14, 21, and 28, and the removed buffer was frozen (−20°C) for drug analysis. AngII concentrations in media were quantified using a radioimmunoassay (RIA) as described below, whereas sacubitril and valsartan were quantified by High performance liquid chromatography (HPLC) with UV detection (267 nm). Briefly, standard curves of each drug were prepared in mobile phase (acetonitrile/methanol, water, 3:5.2, pH 3.0). Isocratic elution (flow rate, 0.5 ml/min) was performed using a C_18_ column (250 × 4.6 mm, 5 µm; ACE Equivalence, EQV-5C18-2546) after drug injection (20 µl). A representative chromatogram is illustrated in Supplemental Fig. 1A. Release rates were calculated based on the drug concentration and the mean flow rate of the osmotic minipumps (5.52 µl/day) and are as follows: sacubitril, 244 µg/day; valsartan, 10.9 µg/day; AngII, 39.2 µg/day.

#### Measurement of Systolic Blood Pressure.

Systolic blood pressure was quantified on conscious mice using a Visitech system (BP-2000; Apex, NC) as described previously (Daugherty et al., 2000; Yiannikouris et al., 2012). Blood pressure of each mouse was quantified the week prior to pump implantation for drug pretreatment and then again during the 3rd week of AngII/drug infusion. Measurements were recorded for 4 days at the same time of day. The first day of measurement was used to acclimate mice to the system, and measurements from each mouse were averaged over the other 3 days of recording (and only mice who had three successful recordings were included in data for statistical analysis).

#### AAA Quantification.

Ultrasound (Vevo 2100 high-resolution imaging system; 55-MHz probe, VisualSonics, Inc.) was performed on anesthetized (2%–3% v/v isoflurane) *Ldlr*^*−/−*^ mice to quantify aortic lumen diameters of the suprarenal abdominal aorta on days 0, 7, 14, and 27 of AngII/drug infusion (Alsiraj et al., 2018; Stegbauer et al., 2019). Abdominal hair was removed by shaving and applying a depilatory cream to anesthetized mice. Lumen diameter measurements were made by two observers using Vevo Laboratory 1.7.2 software. Mice that completed 28 days of AngII infusion were included in measurements of abdominal aortic lumen diameter. Maximal ex vivo abdominal (AAA) diameters were quantified on cleaned, excised abdominal aortas at study endpoint using images obtained with a Nikon SMZ800 dissecting microscope (Alsiraj et al., 2017, 2018). Image analysis was performed using Nikon Elements version 5.11 software. AAA incidence was defined at study endpoint by visual inspection of cleaned aortas by two observers blinded to the experimental groups. Mice that died of a confirmed aortic rupture were included in the calculation of AAA incidence.

#### En Face Quantification of Atherosclerosis.

Atherosclerosis was quantified en face in the aortic arch (AlSiraj et al., 2019). Mice that completed 28 days of AngII infusion were included in the quantification of atherosclerosis. Briefly, the entire length of the aorta was removed, and the intimal surface was exposed, in its entirety, by a longitudinal cut through the inner curvature of the aortic arch and down the anterior aspect of the remaining aorta. A cut was also made through the greater curvature of the aortic arch to the subclavian branch. The tissue was then pinned to a dark surface. Arch areas were defined by drawing a 3-mm line from the left subclavian artery. The intimal area of the aortic arch was defined as the region from the orifice of innominate artery to the orifice of the left subclavian artery. Thoracic areas were defined by drawing a 9-mm line from the end of the arch area to the diaphragm muscle. Lesions were visualized by staining with Oil Red O. Atherosclerotic areas were quantified by drawing a line around the borders and summing the total area of each lesion. Summed lesions were divided by the total arch area to calculate the percent lesion area. Measurements were performed using Nikon NIS-Elements version 5.11.

#### Serum and Plasma Components.

Sera total cholesterol and triglyceride concentrations were quantified from *Ldlr*^*−/−*^ mice that completed 28 days of AngII infusion using enzymatic assay kits (total cholesterol, 999–02601; FUJIFILM Wako Diagnostics USA; triglyceride, l-type TG 994–02891). Plasma renin concentrations were quantified as described previously (Alsiraj et al., 2018). Briefly, mouse plasma (8 *μ*l, collected in the presence of aprotonin 500 KIU/ml and EDTA, 5 mM) was incubated with and without the renin substrate (rat angiotensinogen purified from nephrectomized rat plasma) for 30 minutes. Assay buffer consisted of 100 mM Tris/HCl, pH 8.5, 20 mM EDTA, and 1 mM Phenylmethylsulfonyl fluoride (PMSF). Renin in the mouse plasma converts rat angiotensinogen to angiotensin I (AngI). The amount of AngI formed during the reaction was quantified using an AngI ELISA (IBL, IB59131; analytical sensitivity of 0.14 ng/ml). The amount of AngI formed during the reaction without substrate (background) was subtracted from the amount formed with substrate present for each plasma sample to determine plasma renin concentrations. Plasma AngII concentrations were quantified by RIA with a competitive assay using ^125^I-labeled AngII and a polyclonal AngII (T-4005; Peninsula Laboratories International, Inc.) antibody exhibiting minimal crossreactivity to angiotensin I (0.5%) but 100% crossreactivity to AngIII. The sensitivity of the RIA was 2.5 pg/100 *μ*l.

#### NEP Immunostaining of Human AAAs.

Normal human aorta and AAA paraffin-embedded tissue sections [kindly provided by Dr. John Curci, Washington University, St. Louis; tissue sections were deidentified with only information on age and gender (Thatcher et al., 2014)] were incubated with xylene (100%) and dehydrated with alcohol followed by treatment with Redusol (2 minutes, 40°C). Tissue sections were then treated with automation buffer (1 minute, room temperature), followed by antigen retrieval using a Tris-EDTA buffer and steam (10 minutes) and then a cool-down period (10 minutes). Sections were then washed four times with automation buffer (1 minute), treated with hydrogen peroxide (1% v/v in methanol; 3 minutes), and washed again with automation buffer four times. A blocking agent (5% goat serum) was incubated with sections for 10 minutes at 40°C. Sections were incubated (30 minutes, 40°C) with primary antibody (1:200, anti-neprilysin rabbit polyclonal antibody, AB5458; Millipore) or a biotinylated goat IgG negative control antibody, and washed with automation buffer and then with secondary goat anti-rabbit HRP antibody (1:200; 30 minutes, 40°C, BA-1000; Vector Laboratories). To control for specificity of immunostaining, sections were incubated with secondary antibody only. After washing, sections were incubated with avidin biotin complex (ABC) kit (PK-6100; Vector Laboratories) for avidin-biotin conjugation for 30 minutes at room temperature. Sections were rinsed, and a peroxidase enhancer step was used as a subsequent wash (GTX8279; GeneTex). Finally, an 3-amino-9-ethylcarbazole (AEC) kit (SK-4200; Vector Laboratories) was used for detection of antibody-peroxidase complexes (20 minutes, 37°C), and sections were counterstained with hematoxylin. Glycerol-gelatin (,GG-15 ml; Sigma) was heated and used as a mounting medium. No ethical approval or patient consent was required for these studies.

#### Statistical Analysis.

For illustration, data are presented as means ± S.D. Data sets were tested for normality (Brown-Forsythe) and equal variance (Bartlett’s test). If data did not pass normality, nonparametric tests were used (Kruskal-Wallis test). To define differences between groups (normally distributed data), data were analyzed using unpaired Student’s *t* test for two sample comparisons, or by one-way ANOVA for multiple group comparisons using Holm-Sidak post hoc tests. For categorical data (aneurysm incidence), data were analyzed using Fisher’s exact test. Statistical significance was defined as *P* < 0.05. Statistical analyses were performed using GraphPad Prism 8.3.

## Results

### 

#### NEP mRNA Abundance in Mouse Aortas from AngII-Infused Mice and Immunostaining in Human AAA Tissue Sections.

**Fig. 1.**
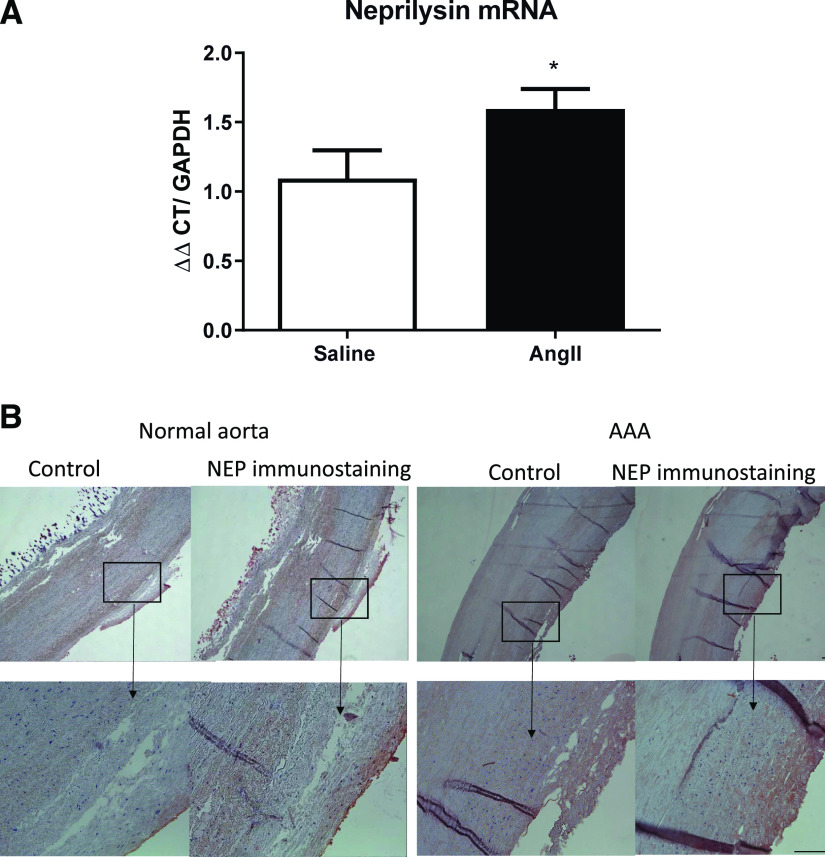
Aortic neprilysin (NEP) mRNA abundance and immunostaining. (A) Male *Ldlr*^*−/−*^ mice fed a Western diet were infused with saline or AngII for 5 days. NEP mRNA abundance in abdominal aorta was increased by AngII compared with saline controls. Data are expressed as means ± S.D. (*n* = 5 mice per group). *P < 0.05 by Student’s *t* test. (B) NEP immunostaining in normal and AAA aorta tissue sections. Control aortic sections were incubated with secondary antibody only. Box indicates magnified area in bottom panel for each section. Scale bar, 100 µm. CT, cycle threshhold.

We infused male *Ldlr*^*−/−*^ mice (fed a Western diet) with saline or AngII for 5 days and harvested abdominal aortas to quantify NEP mRNA abundance. Infusion of AngII resulted in a significant increase in NEP mRNA abundance in abdominal aortas compared with saline-infused controls (Fig. 1A, *P* < 0.05). We immunostained human normal aorta and AAA tissue sections for NEP. Positive NEP immunostaining was detected across the normal aortic wall of human tissue sections, with heavy NEP immunostaining in the intima and adjacent media of human AAA tissue sections (Fig. 1B).

#### Validation of Osmotic Minipump Release of Valsartan and Sacubitril with AngII.

To confirm effective release of drugs from osmotic minipumps, we tested release of valsartan (0.5 mg/kg per day) and sacubitril (9 mg/kg per day) with AngII (1000 ng/kg per minute) from osmotic minipumps over 28 days of delivery. Valsartan (Supplemental Fig. 1B), sacubitril (Supplemental Fig. 1C). and AngII (Supplemental Fig. 1D) were released from osmotic minipumps at the anticipated release rates for each agent (dotted horizontal line of Supplemental Fig. 1, B–D) over the period of delivery examined. For sacubitril, we also tested release rates for a higher drug concentration (15 mg/kg per day), which was not released effectively from osmotic minipumps over a 12-day period (Supplemental Fig. 1E, two different osmotic minipumps examined).

#### Effects of Valsartan and Sacubitril, Alone or in Combination, on Characteristics of AngII-Infused Mice.

Compared with vehicle controls infused with AngII, mice coinfused with AngII plus valsartan (1 or 6 mg/kg per day) or the higher-dose combination of valsartan and sacubitril (0.5/9 mg/kg per day, respectively) had significantly higher body weights (Table 1; *P* < 0.05). We quantified weights (normalized as a percentage of body weight) of heart and kidney as cardiovascular organs that are influenced by infusion of AngII. Heart weights (percentages) were significantly decreased in a dose-dependent manner by valsartan, whereas sacubitril had no effect on heart weight at lower doses, with a significant decrease in heart weight compared with vehicle control at the highest sacubitril dose (9 mg/kg per day) (Table 1; *P* < 0.05). Combination therapy with valsartan and sacubitril had no additional effect on heart weight beyond those observed with valsartan alone (Table 1; *P* > 0.05). With the exception of the lowest dose of valsartan administered (0.3 mg/kg per day), which significantly decreased kidney weights, neither valsartan nor sacubitril significantly influenced kidney weights alone or in combination (Table 1; *P* > 0.05).

**Table 1:** General characteristics of mice from each drug treatment group

Groups	Body Weight	Aorta	Heart	Kidney
	*g*	*%*		
Vehicle	27.4 ± 2.4	0.23 ± 0.07	0.60 ± 0.13	1.14 ± 0.08
0.3 valsartan	26.2 ± 1.1	0.18 ± 0.07	0.60 ± 0.10	1.10 ± 0.05*
0.5 valsartan	28.5 ± 1.9	0.20 ± 0.05	0.50 ± 0.09	1.10 ± 0.08
1 valsartan	30.4 ± 3.5*	0.18 ± 0.04	0.45 ± 0.07*	1.12 ± 0.06
6 valsartan	30.7 ± 1.3*	0.14 ± 0.02*	0.44 ± 0.04*	1.17 ± 0.08
20 valsartan	30.0 ± 2.7	0.11 ± 0.02*	0.42 ± 0.07*	1.20 ± 0.09
1 sacubitril	26.6 ± 2.7	0.22 ± 0.08	0.66 ± 0.10	1.16 ± 0.08
6 sacubitril	29.2 ± 2.0	0.17 ± 0.05	0.63 ± 0.14	1.11 ± 0.06
9 sacubitril	28.0 ± 2.3	0.24 ± 0.070	0.43 ± 0.05*	1.04 ± 0.13
0.3 Val+ 1 Sac	26.8 ± 0.8	0.19 ± 0.04	0.57 ± 0.06	1.20 ± 0.04
0.5 Val+ 9 Sac	30.6 ± 3.0*	0.18 ± 0.05	0.45 ± 0.07*	1.06 ± 0.07

Tissue weights are expressed as a percentage of body weight. Data are means ± S.D.

^*^ *P* < 0.05 compared with vehicle.

#### Effects of Valsartan and Sacubitril, Alone or in Combination, on the Systemic Renin-Angiotensin System and Blood Pressure of AngII-Infused Mice.

**Fig. 2.**
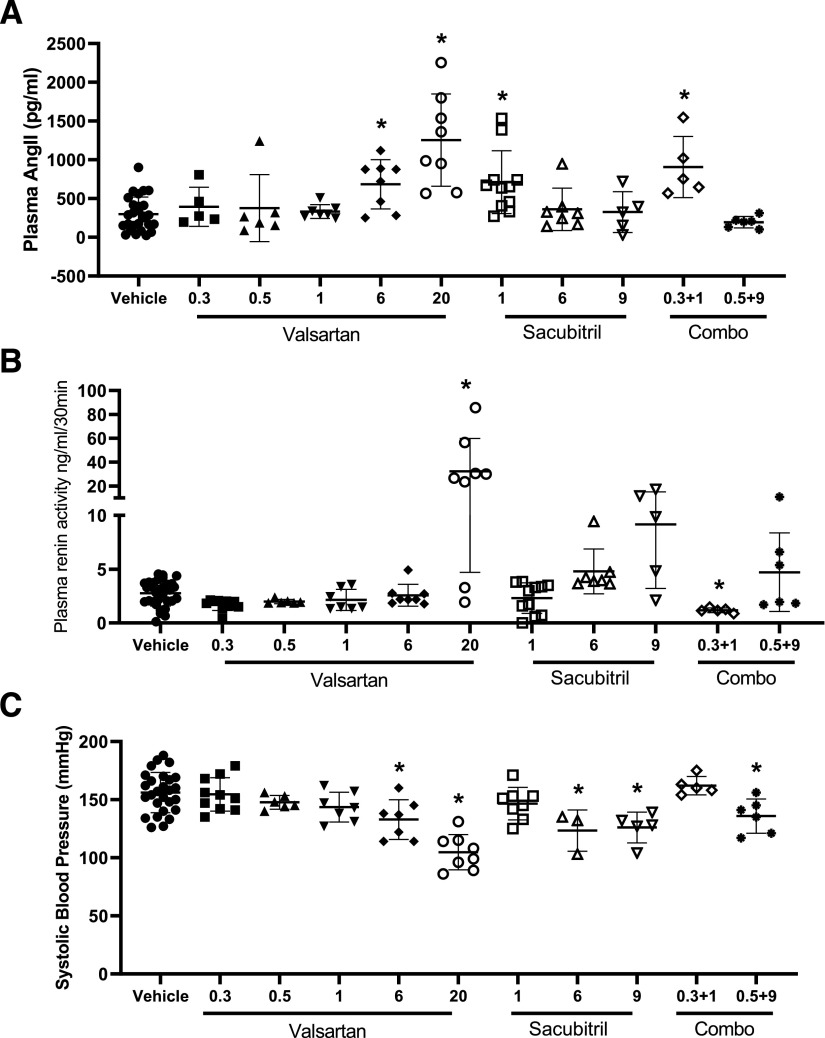
Plasma AngII (A), renin (B), and systolic blood pressures (C) of mice infused with AngII (1000 ng/kg per minute) in the absence (vehicle) or presence of valsartan (0.3, 0.5, 1, 6, or 20 mg/kg per day), sacubitril (1, 6, or 9 mg/kg per day), or combinations thereof (0.3/1; 0.5/9 valsartan/sacubitril). (A) Plasma AngII concentrations increased significantly at higher doses of valsartan. Sacubitril had no effect on plasma AngII concentrations, and the drug combinations tested did not differ from effects observed with either drug alone. (B) Plasma renin concentrations increased significantly at higher doses of valsartan or sacubitril and remained evident when drugs were administered in combination. (C) Systolic blood pressures decreased significantly with higher valsartan or sacubitril doses, but the effectiveness of sacubitril (9 mg/kg per day) to decrease blood pressure was not evident when combined with valsartan (0.5 mg/kg per day). Data are individual mice with means ± S.D. indicated by horizontal lines from vehicle (total of 51 mice, of which 31 mice survived the infusion protocol), valsartan 0.3 (total of 19 mice, of which measurements were obtained from *n* = 5 mice), valsartan 0.5 (total of eight mice, of which six mice survived the infusion protocol), valsartan 1 (total of eight mice, of which seven mice survived the infusion protocol), valsartan 6 (total of eight mice, of which eight mice survived the infusion protocol), valsartan 20 (total of eight mice, of which eight mice survived the infusion protocol), sacubitril 1 (total of 19, of which 11 mice survived the infusion protocol), sacubitril 6 (total of 10, of which seven mice survived the infusion protocol), valsartan 0.3/sacubitril 1 (total of nine mice, of which five mice survived the infusion protocol), valsartan 0.5/sacubitril 9 (total of eight mice, of which six mice survived the infusion protocol). *P < 0.05 compared with vehicle by one-way ANOVA and Kruskal-Wallis test. Closed circle: vehicle closed square: valsartan 0.3 mg/kg/day closed triangle: valsartan 0.5 mg/kg/day closed inverted triangle: valsartan 1 mg/kg/day closed diamond: valsartan 6 mg/kg/day open circle: valsartan 20 mg/kg/day open square: sacubitril 1 mg/kg/day open triangle: sacubitril 6 mg/kg/day open inverted triangle: sacubitril 9 mg/kg/day open diamond: valsartan 0.3 mg/kg/day and sacubitril 1 mg/kg/day closed diamond: valsartan 0.5 mg/kg/day and sacubitril 9 mg/kg/day

It is generally well known that AT1R antagonists remove negative feedback of AngII on renin release from the kidney, resulting in an increase in plasma renin concentrations (Schweda et al., 2007). This effect of AT1R antagonism to increase plasma renin could result in elevations of plasma AngII concentrations, which are generally not of concern with effective AT1R antagonism. AngII is a substrate of NEP, which degrades the peptide into two inactive products (Erdös and Skidgel, 1990), and thus, NEP inhibition might be anticipated to elevate plasma AngII concentrations, which could exacerbate cardiovascular diseases such as hypertension. Administration of valsartan resulted in a dose-dependent elevation of plasma AngII concentrations compared with vehicle (Fig. 2A; vehicle: 298 ± 220, *n* = 27; valsartan 0.3: 394 ± 252, *n* = 5; valsartan 0.5: 376 ± 252, *n* = 6; valsartan 1: 332 ± 88, *n* = 7; valsartan 6: 684 ± 317, *n* = 8; valsartan 20: 1, 254 ± 596 pg/ml, *n* = 8; *P* < 0.05). Interestingly, at low sacubitril concentrations (1 mg/kg per day), plasma AngII concentrations were doubled compared with vehicle, but this effect was not observed at higher sacubitril doses (Fig. 2A; sacubitril 1: 717 ± 405 pg/ml, *n* = 11; sacubitril 9: 326 ± 264 pg/ml, *n* = 5). Elevations of plasma AngII concentrations at 1 mg/kg per day of sacubitril remained evident when combined with valsartan (Fig. 2A; valsartan 0.3/sacubitril 1: 906 ± 396 pg/ml, *n* = 5; *P* < 0.05). Similarly, valsartan administration (20 mg/kg per day) increased plasma renin concentrations (Fig. 2B; vehicle: 2.8 ± 1.2, *n* = 31; valsartan 0.3: 1.7 ± 0.5, *n* = 10; valsartan 0.5: 2.0 ± 0.2, *n* = 6; valsartan 1: 2.1 ± 0.9, *n* = 7; valsartan 6: 2.7 ± 1.0, *n* = 8; valsartan 20: 32.3 ± 27.5 ng of angiotensin I per milliliter per 30 minutes, *n* = 8; *P* < 0.05). Despite elevations of plasma AngII and renin concentrations, valsartan resulted in a dose-dependent reduction of systolic blood pressures of AngII-infused mice (Fig. 2C; vehicle: 156 ± 17, *n* = 27; valsartan 0.3: 155 ± 14, *n* = 10; valsartan 0.5: 148 ± 6, *n* = 6; valsartan 1: 144 ± 13, *n* = 6; valsartan 6: 133 ± 17, *n* = 7; valsartan 20: 105 ± 15 mm Hg, *n* = 8; *P* < 0.05). At the lower dose (1 mg/kg per day) of sacubitril, there was no significant effect on systolic blood pressures, whereas higher doses of sacubitril significantly reduced systolic blood pressures (Fig. 2C; sacubitril 1: 147 ± 14, *n* = 8; sacubitril 6: 123 ± 18, *n* = 5; sacubitril 9: 126 ± 13 mm Hg, *n* = 5; *P* < 0.05). The reduction in systolic blood pressure at 9 mg/kg per day of sacubitril remained evident when combined with a dose (0.5 mg/kg per day) of valsartan that alone had no effect on systolic blood pressure (Fig. 2C; valsartan 0.3/sacubitril 9: 136 ± 15 mm Hg, *n* = 6; *P* < 0.05).

#### Effects of Valsartan and Sacubitril, Alone or in Combination, on AngII-Induced AAAs.

**Fig. 3.**
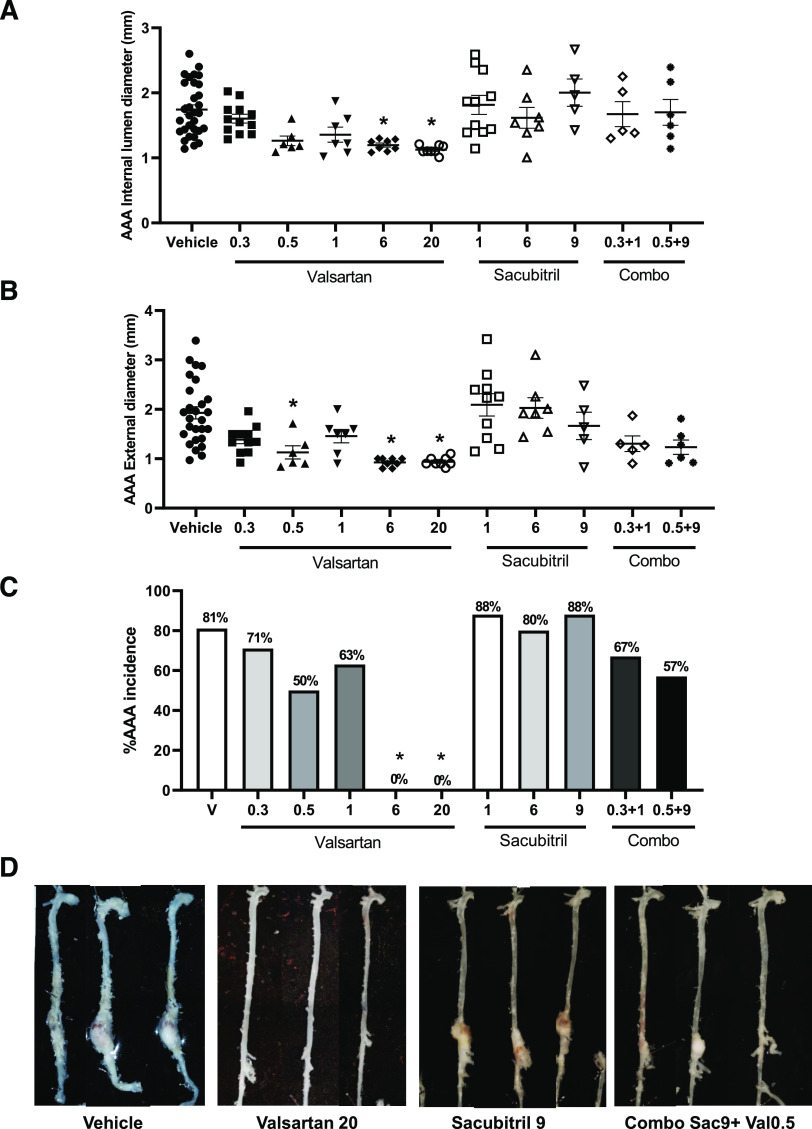
Effects of valsartan and sacubitril, alone or in combination, on AngII-induced AAAs. Mice were infused with AngII (1000 ng/kg per minute) in the absence (vehicle) or presence of valsartan (0.3, 0.5, 1, 6, or 20 mg/kg per day), sacubitril (1, 6, or 9 mg/kg per day), or combinations thereof (0.3/1; 0.5/9 valsartan/sacubitril). (A) Abdominal aortic internal lumen diameters (day 28 of infusions) were decreased dose-dependently by valsartan, whereas sacubitril had no effect alone or in combination with valsartan. (B) AAA external diameters were decreased dose-dependently by valsartan, with no effect of sacubitril alone or in combination with valsartan. For (A) and (B), data are individual mice with means ± S.D. indicated by horizontal lines from vehicle (total of 51 mice, of which 31 mice survived the infusion protocol), valsartan 0.3 (total of 19 mice, of which 11 mice survived the infusion protocol), valsartan 0.5 (total of eight mice, of which six mice survived the infusion protocol), valsartan 1 (total of eight mice, of which seven mice survived the infusion protocol), valsartan 6 (total of eight mice, of which eight mice survived the infusion protocol), valsartan 20 (total of eight mice, of which eight mice survived the infusion protocol), sacubitril 1 (total of 19, of which 11 survived the infusion protocol), sacubitril 6 (total of 10, of which seven mice survived the infusion protocol), valsartan 0.3/sacubitril 1 (total of nine mice, of which five mice survived the infusion protocol), and valsartan 0.5/sacubitril 9 (total of eight mice of which six mice survived the infusion protocol). (C) AAA incidence (percentage) was decreased dose-dependently by valsartan, with no effect of sacubitril alone or in combination with valsartan. Data are individual mice with means ± S.D. indicated by horizontal lines from vehicle (*n* = 51), valsartan 0.3 (*n* = 19), valsartan 0.5 (*n* = 8), valsartan 1 (*n* = 8), valsartan 6 (*n* = 8), valsartan 20 (*n* = 8), sacubitril 1 (*n* = 19), sacubitril 6 (*n* = 10), valsartan 0.3/sacubitril 1 (*n* = 9), and valsartan 0.5/sacubitril 9 (*n* = 8). (D) Representative aortas from mice of each treatment group. *P < 0.05 compared with vehicle by one-way ANOVA, Kruskal-Wallis, or Fisher’s exact test.Sacubitril, Sac; Valsartan, Val.

Valsartan resulted in a dose-dependent reduction of internal abdominal aortic lumen diameters (Fig. 3A, 28 days of infusion; vehicle: 1.7 ± 0.4, *n* = 31; valsartan 0.3: 1.6 ± 0.2, *n* = 12; valsartan 0.5: 1.3 ± 0.2, *n* = 6; valsartan 1: 1.4 ± 0.3, *n* = 7; valsartan 6: 1.2 ± 0.1, *n* = 8; valsartan 20: 1.1 ± 0.1 mm, *n* = 8; *P* < 0.05), maximal AAA external diameters (Fig. 3B; vehicle: 1.9 ± 0.6, *n* = 27; valsartan 0.3: 1.4 ± 0.3, *n* = 11; valsartan 0.5: 1.1 ± 0.3, *n* = 7; valsartan 1: 1.5 ± 0.4, *n* = 7; valsartan 6: 0.9 ± 0.1, *n* = 8; valsartan 20: 0.9 ± 0.1 mm, *n* = 8; *P* < 0.05), and AAA incidence (Fig. 3, C and D; *P* < 0.05). In contrast, sacubitril had no significant effect on any of these parameters of AngII-infused mice (Fig. 3, A–C; *P* > 0.05). Moreover, the combination drug doses tested did not result in further reductions of AngII-induced AAAs than did administration of valsartan alone, and for some parameters (maximal AAA external diameters), significant reductions by valsartan (0.5 mg/kg per day) were no longer evident when combined with sacubitril (Fig. 3, A and B).

#### Effects of Valsartan and Sacubitril, Alone or in Combination, on Serum Cholesterol Concentrations, Serum Triglyceride Concentrations, and Atherosclerosis of AngII-Infused Mice.

**Fig. 4.**
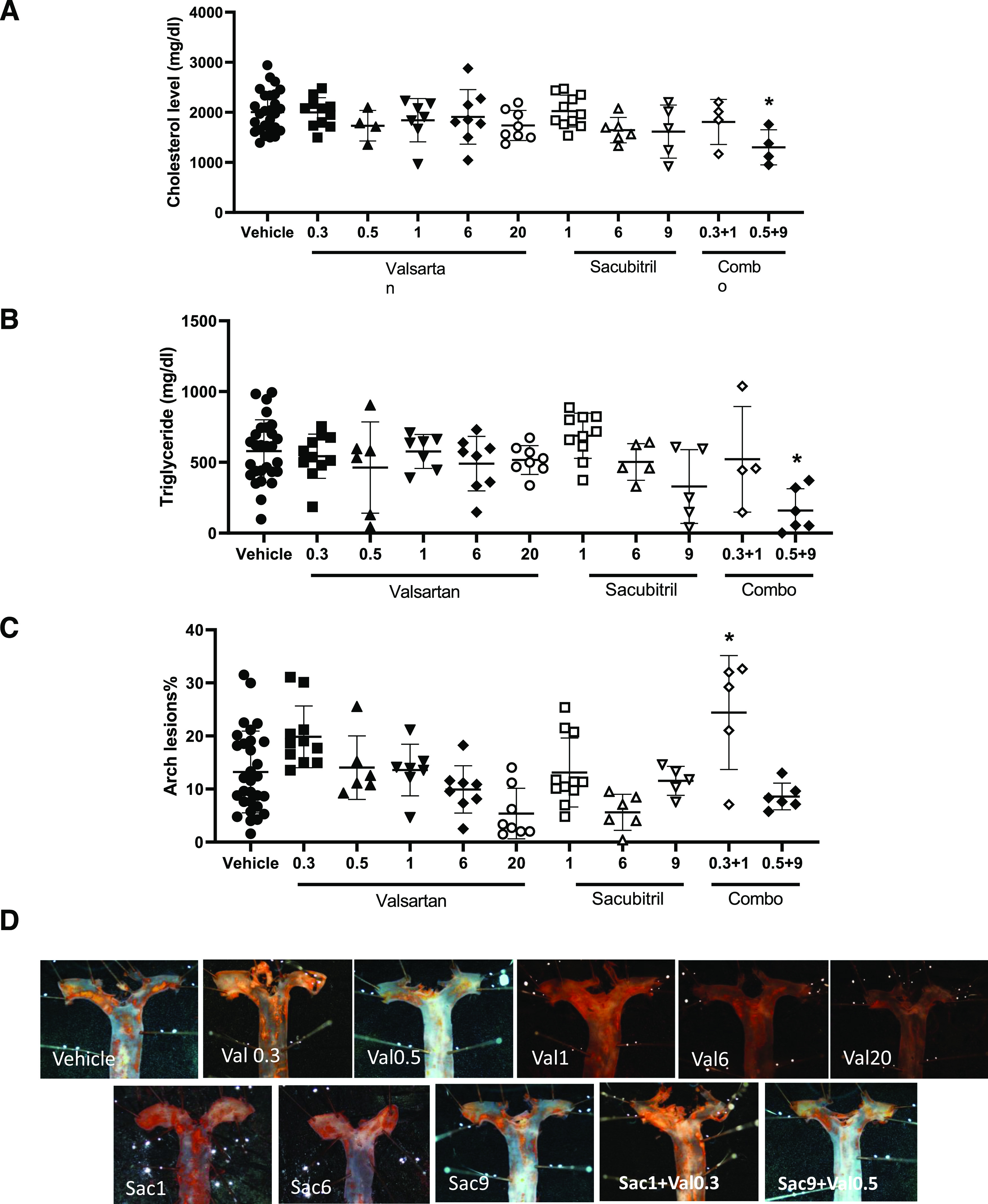
Effects of valsartan and sacubitril, alone or in combination, on serum lipids and atherosclerotic lesion surface areas of AngII-infused mice. Mice were infused with AngII (1000 ng/kg per minute) in the absence (vehicle) or presence of valsartan (0.3, 0.5, 1, 6, or 20 mg/kg per day), sacubitril (1, 6, or 9 mg/kg per day), or combinations thereof (0.3/1; 0.5/9 valsartan/sacubitril). (A) Serum cholesterol concentrations were not influenced by valsartan or sacubitril alone. However, when drugs were combined at the higher dose (0.5/9 valsartan/sacubitril), serum cholesterol decreased significantly. (B) Serum triglyceride concentrations were not influenced by valsartan or sacubitril alone. However, when drugs were combined at the higher dose (0.5/9 valsartan/sacubitril), serum triglyceride decreased significantly. (C) Valsartan or sacubitril had no significant effect on atherosclerosis in the aortic arch (percent lesion surface area) when administered alone. However, the lower-dose drug combination (0.3/1, valsartan/sacubitril) significantly increased atherosclerosis. (D) Oil Red O–stained representative aortas from mice of each treatment group. For (A–C), data are individual mice with means ± S.D. indicated by horizontal lines from vehicle (total of 51 mice, of which 31 mice survived the infusion protocol), valsartan 0.3 (total of 19 mice, of which 11 mice survived the infusion protocol), valsartan 0.5 (total of eight mice, of which six mice survived the infusion protocol), valsartan 1 (total of eight mice, of which seven mice survived the infusion protocol), valsartan 6 (total of eight mice, of which eight mice survived the infusion protocol), valsartan 20 (total of eight mice, of which eight mice survived the infusion protocol), sacubitril 1 (total of 19, of which 11 survived the infusion protocol), sacubitril 6 (total of 10, of which seven mice survived the infusion protocol), valsartan 0.3/sacubitril 1 (total of nine mice, of which five mice survived the infusion protocol), and valsartan 0.5/sacubitril 9 (total of eight mice, of which six mice survived the infusion protocol). *P < 0.05 compared with vehicle by one-way ANOVA.

There were no significant effects of valsartan or sacubitril on serum cholesterol or triglyceride concentrations of AngII-infused mice (Fig. 4, A and B; *P* > 0.05). However, at the higher-dose combination of both drugs, serum cholesterol (vehicle: 2007 ± 412, *n* = 27; valsartan 0.5/sacubitril 9: 1301 ± 351 mg/dl, *n* = 4) and triglyceride concentrations (vehicle: 579 ± 221; valsartan 0.5/sacubitril 9: 159 ± 54 mg/dl, *n* = 6) were significantly reduced compared with vehicle (Fig. 4, A and B; *P* < 0.05). Infusion of AngII was demonstrated to increase the extent of atherosclerosis in hypercholesterolemic mice (Daugherty et al., 2000), which was abolished by the AT1R antagonist losartan (Daugherty et al., 2001). Similarly, in this study valsartan resulted in a dose-dependent reduction in the percent lesion surface area of the aortic arch of AngII-infused mice (Fig. 4, C and D; vehicle: 13.2 ± 7.7, *n* = 30; valsartan 0.3: 19.8 ± 5.8, *n* = 11; valsartan 0.5: 14.0 ± 6.0, *n* = 6; valsartan 1: 13.6 ± 4.9, *n* = 7; valsartan 6: 9.9 ± 4.5, *n* = 8; valsartan 20: 5.4 ± 4.8% lesion surface area). However, effects of valsartan on atherosclerotic lesion formation were not significant. Moreover, sacubitril had no significant effect on percent atherosclerotic lesion surface area when administered alone (Fig. 4C; *P* > 0.05). Paradoxically, at the lower drug combination dose examined (0.3 valsartan/1 mg/kg per day sacubitril: 24.4 ± 10.7, *n* = 5) there was a significant increase in atherosclerotic lesion surface area compared with vehicle, even though neither sacubitril nor valsartan at these doses influenced AngII-induced atherosclerosis when administered as individual agents (Fig. 4C; *P* < 0.05).

## Discussion

We investigated the dose-dependent therapeutic effectiveness of AT1R antagonism alone or in combination with NEP inhibition on AngII-induced cardiovascular responses of hypertension, atherosclerosis, and AAA formation in hypercholesterolemic mice. The impetus for this investigation was manifold and included varying roles of NEP substrates/products in the development of hypertension, atherosclerosis, or AAAs; the potential for alterations in systemic AngII concentrations upon NEP inhibition alone; and the possibility of additive or synergistic effectiveness of AT1R antagonism when combined with NEP inhibition in several AngII-induced cardiovascular diseases. Results from this study demonstrate that cardiovascular diseases of hypertension, atherosclerosis, or AAAs induced by infusion of AngII are attenuated by AT1R antagonism. In contrast, although sacubitril at higher doses reduced AngII-induced hypertension, the drug had minimal benefits on other AngII-induced responses when administered alone and did not improve effectiveness of AT1R antagonism in combination therapy. Moreover, depending on the dose of sacubitril and its effects on circulating AngII concentrations, our results do not support NEP inhibition in the absence of sufficient AT1R blockade as an effective therapeutic approach for AngII-induced atherosclerosis.

In this study, we performed dose-response studies with both valsartan and sacubitril, alone or in two different drug combinations, on AngII-induced hypertension, atherosclerosis, and AAA formation in hypercholesterolemic mice. As AT1R antagonism has been examined extensively against each of these AngII-induced cardiovascular diseases in experimental models, our rationale was to determine whether sacubitril would provide additive or synergistic benefits at doses of valsartan that did not have extensive effects when administered alone. In addition, we contrasted this combination dose strategy against a higher valsartan dose (0.5 mg/kg per day) that had some degree of efficacy to reduce AngII-induced cardiovascular diseases. Using this approach, we were unable to demonstrate added benefit of sacubitril beyond effects of valsartan alone on any of these AngII-induced responses. However, we did observe a significant effect of the higher dose drug combination to reduce serum cholesterol and triglyceride concentrations.

To our knowledge, this is the first report of AngII-stimulated NEP mRNA abundance and the presence of positive NEP immunostaining in normal or aneurysmal human AAA tissue. However, there is increasing evidence that NEP substrates and/or products, including brain or natriuretic peptides, may serve as biomarkers or prognostic indicators after surgery of human AAAs (Rajagopalan et al., 2008; Vetrugno et al., 2012; Bryce et al., 2013; Folsom et al., 2015; Acosta et al., 2018; Chan et al., 2019). Given these findings, it is surprising that NEP inhibition with sacubitril had no significant effects on AngII-induced AAA formation. In contrast, similar to previous findings (Daugherty et al., 2001; Iida et al., 2012), AT1R antagonism with valsartan was highly effective at reducing the formation of AngII-induced AAAs. It is possible that NEP inhibition may be effective in other experimental AAA models that do not involve AngII as the initiating stimulus, since NEP inhibition at some doses (1 mg/kg per day) elevated circulating AngII concentrations that would be anticipated to worsen AngII-induced AAAs. Unlike congestive heart failure, our results do not support additive or synergistic benefit of combined therapy with AT1R antagonism and NEP inhibition to reduce experimental AngII-induced AAA formation.

Similar to AAAs, evolving evidence indicates a role for NEP in atherosclerotic lesion formation. Specifically, oral inhibition of neutral endopeptidase (NEP) suppressed atherosclerotic lesion formation in cholesterol diet–fed New Zealand white rabbits (Kugiyama et al., 1996; Grantham et al., 2000). Moreover, a dual inhibitor of angiotensin converting enzyme and NEP (omapatrilat) reduced fatty streak or atherosclerotic lesion formation in apolipoprotein E (ApoE)-deficient (Arnal et al., 2001) or *Ldlr*^*−/−*^ mice (Levy et al., 2003). Similar effects were observed in hyperlipidemic rabbits administered ramipril (ACE inhibitor) and an NEP inhibitor (AVE 7688) (Weckler et al., 2003). More recently, a sacubitril/valsartan complexed drug combination (LCZ696) was found to decrease lipid deposition in carotid artery histologic sections from ApoE^−/−^ mice fed a Western diet (Zhang et al., 2019). These effects were associated with elevations of plasma concentrations of brain natriuretic peptide and reductions in circulating inflammatory factors (e.g., interleukin-6, matrix metalloproteinase-8) of mice administered LCZ696 compared with valsartan alone. Our results do not support added benefits of sacubitril administration beyond those observed with valsartan alone on AngII-induced atherosclerotic lesion formation. This result was surprising given that the high-dose combination of valsartan/sacubitril did result in a significant reduction in serum lipids, which were observed in mice experiencing increased body weights compared with vehicle controls. Reductions in serum lipids such as plasma cholesterol or triglyceride concentrations typically reduce the development of atherosclerosis. Rather, we observed potential harmful effects of the lower-dose drug combination to increase atherosclerosis. The increase in atherosclerosis of mice administered the lower-dose drug combination in the present study was observed in mice experiencing elevated plasma AngII concentrations, which may have contributed to this finding. Differences between results of this study and previous studies (Zhang et al., 2019) include the choice of drug [combined inhibition through complexed LCZ696 (Zhang et al., 2019) vs. individual drug NEP inhibition/AT1R antagonism of the current study], route of administration [daily oral gavage (Zhang et al., 2019) vs. continuous subcutaneous drug infusion of the current study], and mouse genetic background [ApoE^−/−^ (Zhang et al., 2019) vs. *Ldlr*^*−/−*^ mice of the current study]. Moreover, our results examining combined NEP inhibition and AT1R antagonism focus on atherosclerosis induced by infusion of AngII, which may differ from atherosclerosis in response to other stimuli (e.g., diet or ApoE deficiency).

In contrast to atherosclerosis or AAA formation, in this study administration of sacubitril (6 or 9 mg/kg per day) decreased AngII-induced elevations of systolic blood pressure. However, there were no added benefits of sacubitril (9 mg/kg per day) on AngII-induced hypertension when combined with valsartan (0.5 mg/kg per day). These results suggest that antihypertensive actions of sacubitril may be unrelated to AngII actions at AT1R to elevate blood pressure, with potential effects of NEP inhibition on other vasoactive NEP substrates (e.g., endothelin, bradykinin).

In the present study, we examined subcutaneous infusion of valsartan with or without sacubitril using osmotic minipumps implanted in AngII-infused mice, as this mode of administration would avoid issues of bioavailability after oral dosing and provide for continuous drug delivery. Since this drug delivery method for sacubitril has not been examined previously, we performed pilot studies assessing the release rate of drugs when combined together in an osmotic minipump for subcutaneous delivery. We found that sacubitril was not effectively released from osmotic minipumps at doses of 15 mg/kg per day. For this reason, we limited the maximum dose of sacubitril to 9 mg/kg per day alone or in combination with valsartan. Our results indicate that a sacubitril dose of 1 mg/kg per day elevated plasma AngII concentrations, suggesting that sacubitril inhibited NEP-mediated degradation of AngII. Moreover, we observed effects of sacubitril (6 or 9 mg/kg per day) to decrease systolic blood pressure and reduce serum cholesterol and triglyceride concentrations (when combined with 0.5 mg/kg per day valsartan), supporting drug effects at the studied doses and route of administration. However, a limitation of this study is that limits on sacubitril solubility within osmotic minipumps did not allow for examination of higher doses of sacubitril that have been more recently tested after oral administration in other experimental cardiovascular models (Croteau et al., 2020; Li et al., 2020; Tam et al., 2020). It is also possible that differences in NEP expression levels between mouse strains may have influenced results of the present study (Korshunov et al., 2019). Moreover, other limitations include the choice of animal model (highly related to AngII as the initiating stimulus), difficulties in extrapolating findings from mice to humans, and relevance of the systemic drug concentrations to those achieved in humans. A limitation of our approach is that, for endpoint measures, we cannot include data from mice that do not survive the entire infusion protocol, limiting the number of experimental mice per group. Finally, although these results do not support the addition of NEP inhibition to AT1R antagonism against experimental AngII-induced hypertension, atherosclerosis, or AAA formation, this drug combination may have benefit against other modes of inducing these cardiovascular diseases.

In conclusion, results from this study do not support additive benefits, beyond those observed with AT1R receptor antagonism alone, of the addition of sacubitril for experimental AngII-induced hypertension, atherosclerosis, or AAA formation.

## Abbreviations

AAA: abdominal aortic aneurysm
AngI: angiotensin I
AngII: angiotensin II
ApoE: apolipoprotein E
AT1R: angiotensin type 1 receptor
*Ldlr*^*−/−*^: low-density lipoprotein receptor deficient
NEP: neprilysin
RIA: radioimmunoassay

## References

[B1] Abassi ZA Tate JE Golomb E Keiser HR (1992) Role of neutral endopeptidase in the metabolism of endothelin. Hypertension 20:89–95. 1618556 10.1161/01.hyp.20.1.89

[B2] Acosta S Gottsäter A Engström G Melander O Zarrouk M Nilsson PM Smith JG (2018) B-type natriuretic peptide for prediction of incident clinically significant abdominal aortic aneurysm: a population-based prospective study. Vasc Med 23:46–51. 29343179 10.1177/1358863X17745150

[B3] AlSiraj Y Chen X Thatcher SE Temel RE Cai L Blalock E Katz W Ali HM Petriello M Deng P (2019) XX sex chromosome complement promotes atherosclerosis in mice. Nat Commun 10:2631. 31201301 10.1038/s41467-019-10462-z PMC6643208

[B4] Alsiraj Y Thatcher SE Blalock E Fleenor B Daugherty A Cassis LA (2018) Sex chromosome complement defines diffuse versus focal angiotensin II-induced aortic pathology. Arterioscler Thromb Vasc Biol 38:143–153. 29097367 10.1161/ATVBAHA.117.310035 PMC5864127

[B5] Alsiraj Y Thatcher SE Charnigo R Chen K Blalock E Daugherty A Cassis LA (2017) Female mice with an XY sex chromosome complement develop severe angiotensin II-induced abdominal aortic aneurysms. Circulation 135:379–391. 27815372 10.1161/CIRCULATIONAHA.116.023789 PMC5470072

[B6] Arnal JF Castano C Maupas E Mugniot A Darblade B Gourdy P Michel JB Bayard F (2001) Omapatrilat, a dual angiotensin-converting enzyme and neutral endopeptidase inhibitor, prevents fatty streak deposit in apolipoprotein E-deficient mice. Atherosclerosis 155:291–295. 11254898 10.1016/s0021-9150(00)00565-7

[B7] Bai HY Mogi M Nakaoka H Kan-No H Tsukuda K Chisaka T Wang XL Kukida M Shan BS Yamauchi T (2015) Pre-treatment with LCZ696, an orally active angiotensin receptor neprilysin inhibitor, prevents ischemic brain damage. Eur J Pharmacol 762:293–298. 26057694 10.1016/j.ejphar.2015.05.059

[B8] Bayes-Genis A Barallat J Richards AM (2016) A test in context: neprilysin: function, inhibition, and biomarker. J Am Coll Cardiol 68:639–653. 27491909 10.1016/j.jacc.2016.04.060

[B9] Benedict CR Johnstone DE Weiner DH Bourassa MG Bittner V Kay R Kirlin P Greenberg B Kohn RM Nicklas JM SOLVD Investigators (1994) Relation of neurohumoral activation to clinical variables and degree of ventricular dysfunction: a report from the Registry of Studies of Left Ventricular Dysfunction. J Am Coll Cardiol 23:1410–1420. 7909822 10.1016/0735-1097(94)90385-9

[B10] Bryce GJ Payne CJ Gibson SC Byrne DS Delles C McClure J Kingsmore DB (2013) B-type natriuretic peptide predicts postoperative cardiac events and mortality after elective open abdominal aortic aneurysm repair. J Vasc Surg 57:345–353. 23058722 10.1016/j.jvs.2012.07.053

[B11] Chan W Kingwell BA Schneider HG Cox G Natoli A Starr J Croft KD Dart AM Duffy SJ (2019) Preoperative biomarker evaluation for the prediction of cardiovascular events after major vascular surgery. J Vasc Surg 70:1564–1575. 31653377 10.1016/j.jvs.2019.02.041

[B12] Croteau D Qin F Chambers JM Kallick E Luptak I Panagia M Pimentel DR Siwik DA Colucci WS (2020) Differential effects of sacubitril/valsartan on diastolic function in mice with obesity-related metabolic heart disease. JACC Basic Transl Sci 5:916–927. 33015414 10.1016/j.jacbts.2020.07.006 PMC7524781

[B13] Daugherty A Cassis L (1999) Chronic angiotensin II infusion promotes atherogenesis in low density lipoprotein receptor -/- mice. Ann N Y Acad Sci 892:108–118. 10842656 10.1111/j.1749-6632.1999.tb07789.x

[B14] Daugherty A Manning MW Cassis LA (2000) Angiotensin II promotes atherosclerotic lesions and aneurysms in apolipoprotein E-deficient mice. J Clin Invest 105:1605–1612. 10841519 10.1172/JCI7818 PMC300846

[B15] Daugherty A Manning MW Cassis LA (2001) Antagonism of AT2 receptors augments angiotensin II-induced abdominal aortic aneurysms and atherosclerosis. Br J Pharmacol 134:865–870. 11606327 10.1038/sj.bjp.0704331 PMC1573019

[B16] Erdös EG Skidgel RA (1989) Neutral endopeptidase 24.11 (enkephalinase) and related regulators of peptide hormones. FASEB J 3:145–151. 2521610

[B17] Erdös EG Skidgel RA (1990) Renal metabolism of angiotensin I and II. Kidney Int Suppl 30:S24–S27. 2175370

[B18] Feringa HH Bax JJ Elhendy A de Jonge R Lindemans J Schouten O van den Meiracker AH Boersma E Schinkel AF Kertai MD (2006) Association of plasma N-terminal pro-B-type natriuretic peptide with postoperative cardiac events in patients undergoing surgery for abdominal aortic aneurysm or leg bypass. Am J Cardiol 98:111–115. 16784932 10.1016/j.amjcard.2006.01.058

[B19] Feringa HH Schouten O Dunkelgrun M Bax JJ Boersma E Elhendy A de Jonge R Karagiannis SE Vidakovic R Poldermans D (2007) Plasma N-terminal pro-B-type natriuretic peptide as long-term prognostic marker after major vascular surgery. Heart 93:226–231. 16914484 10.1136/hrt.2006.093716 PMC1861400

[B20] Ferro CJ Spratt JC Haynes WG Webb DJ (1998) Inhibition of neutral endopeptidase causes vasoconstriction of human resistance vessels in vivo. Circulation 97:2323–2330. 9639376 10.1161/01.cir.97.23.2323

[B21] Folsom AR Yao L Alonso A Lutsey PL Missov E Lederle FA Ballantyne CM Tang W (2015) Circulating biomarkers and abdominal aortic aneurysm incidence: the Atherosclerosis Risk in Communities (ARIC) study. Circulation 132:578–585. 26085454 10.1161/CIRCULATIONAHA.115.016537 PMC4543558

[B22] Grantham JA Schirger JA Wennberg PW Sandberg S Heublein DM Subkowski T Burnett JC Jr (2000) Modulation of functionally active endothelin-converting enzyme by chronic neutral endopeptidase inhibition in experimental atherosclerosis. Circulation 101:1976–1981. 10779465 10.1161/01.cir.101.16.1976

[B23] Gu J Noe A Chandra P Al-Fayoumi S Ligueros-Saylan M Sarangapani R Maahs S Ksander G Rigel DF Jeng AY (2010) Pharmacokinetics and pharmacodynamics of LCZ696, a novel dual-acting angiotensin receptor-neprilysin inhibitor (ARNi). J Clin Pharmacol 50:401–414. 19934029 10.1177/0091270009343932

[B24] Henriques TA Huang J D’Souza SS Daugherty A Cassis LA (2004) Orchidectomy, but not ovariectomy, regulates angiotensin II-induced vascular diseases in apolipoprotein E-deficient mice. Endocrinology 145:3866–3872. 15105380 10.1210/en.2003-1615

[B25] Iida Y Xu B Schultz GM Chow V White JJ Sulaimon S Hezi-Yamit A Peterson SR Dalman RL (2012) Efficacy and mechanism of angiotensin II receptor blocker treatment in experimental abdominal aortic aneurysms. PLoS One 7:e49642. 23226500 10.1371/journal.pone.0049642 PMC3513299

[B26] Ishii M Kaikita K Sato K Sueta D Fujisue K Arima Y Oimatsu Y Mitsuse T Onoue Y Araki S (2017) Cardioprotective effects of LCZ696 (sacubitril/valsartan) after experimental Acute myocardial infarction. JACC Basic Transl Sci 2:655–668. 30062181 10.1016/j.jacbts.2017.08.001 PMC6059351

[B27] Januzzi JL Jr Prescott MF Butler J Felker GM Maisel AS McCague K Camacho A Piña IL Rocha RA Shah AM PROVE-HF Investigators (2019) Association of change in N-terminal pro-B-type natriuretic peptide following initiation of sacubitril-valsartan treatment with cardiac structure and function in patients with heart failure with reduced ejection fraction. JAMA 322:1–11. 10.1001/jama.2019.12821 PMC6724151 31475295

[B28] Koglin J Pehlivanli S Schwaiblmair M Vogeser M Cremer P vonScheidt W (2001) Role of brain natriuretic peptide in risk stratification of patients with congestive heart failure. J Am Coll Cardiol 38:1934–1941. 11738297 10.1016/s0735-1097(01)01672-2

[B29] Korshunov VA Quinn B Faiyaz A Ahmed R Sowden MP Doyley MM Berk BC (2019) Strain-selective efficacy of sacubitril/valsartan on carotid fibrosis in response to injury in two inbred mouse strains. Br J Pharmacol 176:2795–2807. 31077344 10.1111/bph.14708 PMC6609549

[B30] Kugiyama K Sugiyama S Matsumura T Ohta Y Doi H Yasue H (1996) Suppression of atherosclerotic changes in cholesterol-fed rabbits treated with an oral inhibitor of neutral endopeptidase 24.11 (EC 3.4.24.11). Arterioscler Thromb Vasc Biol 16:1080–1087. 8696950 10.1161/01.atv.16.8.1080

[B31] Lei B Nakano D Fan YY Kitada K Hitomi H Kobori H Mori H Masaki T Nishiyama A (2012) Add-on aliskiren elicits stronger renoprotection than high-dose valsartan in type 2 diabetic KKAy mice that do not respond to low-dose valsartan. J Pharmacol Sci 119:131–138. 22673148 10.1254/jphs.12031fp PMC3396743

[B32] Levy Z Dvir A Shaish A Trestman S Cohen H Levkovietz H Rhachmani R Ravid M Harats D (2003) Omapatrilat, an angiotensin-converting enzyme and neutral endopeptidase inhibitor, attenuates early atherosclerosis in diabetic and in nondiabetic low-density lipoprotein receptor-deficient mice. Int J Exp Diabesity Res 4:59–64. 12745671 10.1080/15438600303728 PMC2480500

[B33] Li X Zhu Q Wang Q Zhang Q Zheng Y Wang L Jin Q (2020) Protection of sacubitril/valsartan against pathological cardiac remodeling by inhibiting the NLRP3 inflammasome after relief of pressure overload in mice. Cardiovasc Drugs Ther 34:629–640. 32444995 10.1007/s10557-020-06995-x PMC7497317

[B34] Lillyblad MP (2015) Dual angiotensin receptor and neprilysin inhibition with sacubitril/valsartan in chronic systolic heart failure: understanding the New PARADIGM. Ann Pharmacother 49:1237–1251. 26175499 10.1177/1060028015593093

[B35] Liu J Sawada H Howatt DA Moorleghen JJ Vsevolozhskaya O Daugherty A Lu HS (2020) Hypercholesterolemia accelerates both the initiation and progression of angiotensin II-induced abdominal aortic aneurysms. Ann Vasc Med Res 6:1099. 32432166 PMC7236767

[B36] Lu H Howatt DA Balakrishnan A Graham MJ Mullick AE Daugherty A (2016) Hypercholesterolemia induced by a PCSK9 gain-of-function mutation augments angiotensin II-induced abdominal aortic aneurysms in C57bl/6 mice-brief report. Arterioscler Thromb Vasc Biol 36:1753–1757. 27470509 10.1161/ATVBAHA.116.307613 PMC5001883

[B37] Packer M McMurray JJ Desai AS Gong J Lefkowitz MP Rizkala AR Rouleau JL Shi VC Solomon SD Swedberg K PARADIGM-HF Investigators and Coordinators (2015) Angiotensin receptor neprilysin inhibition compared with enalapril on the risk of clinical progression in surviving patients with heart failure. Circulation 131:54–61. 25403646 10.1161/CIRCULATIONAHA.114.013748

[B38] Park KS Li Y Zhang Y Gerbes AL Liu H Swain MG Lee SS (2003) Effects of the neutral endopeptidase inhibitor thiorphan on cardiovascular and renal function in cirrhotic rats. Br J Pharmacol 139:81–88. 12746226 10.1038/sj.bjp.0705219 PMC1573821

[B39] Rajagopalan S Croal BL Bachoo P Hillis GS Cuthbertson BH Brittenden J (2008) N-terminal pro B-type natriuretic peptide is an independent predictor of postoperative myocardial injury in patients undergoing major vascular surgery. J Vasc Surg 48:912–917; discussion 917. 18586440 10.1016/j.jvs.2008.05.015

[B40] Roksnoer LC van Veghel R Clahsen-van Groningen MC de Vries R Garrelds IM Bhaggoe UM van Gool JM Friesema EC Leijten FP Hoorn EJ (2016) Blood pressure-independent renoprotection in diabetic rats treated with AT1 receptor-neprilysin inhibition compared with AT1 receptor blockade alone. Clin Sci (Lond) 130:1209–1220. 27129187 10.1042/CS20160197

[B41] Roksnoer LC van Veghel R de Vries R Garrelds IM Bhaggoe UM Friesema EC Leijten FP Poglitsch M Domenig O Clahsen-van Groningen MC (2015) Optimum AT1 receptor-neprilysin inhibition has superior cardioprotective effects compared with AT1 receptor blockade alone in hypertensive rats. Kidney Int 88:109–120. 25830765 10.1038/ki.2015.107

[B42] Schirger JA Grantham JA Kullo IJ Jougasaki M Wennberg PW Chen HH Lisy O Miller V Simari RD Burnett JC Jr (2000) Vascular actions of brain natriuretic peptide: modulation by atherosclerosis and neutral endopeptidase inhibition. J Am Coll Cardiol 35:796–801. 10716485 10.1016/s0735-1097(99)00593-8

[B43] Schling P Schäfer T (2002) Human adipose tissue cells keep tight control on the angiotensin II levels in their vicinity. J Biol Chem 277:48066–48075. 12196514 10.1074/jbc.M204058200

[B44] Schweda F Friis U Wagner C Skott O Kurtz A (2007) Renin release. Physiology (Bethesda) 22:310–319. 17928544 10.1152/physiol.00024.2007

[B45] Stegbauer J Thatcher SE Yang G Bottermann K Rump LC Daugherty A Cassis LA (2019) Mas receptor deficiency augments angiotensin II-induced atherosclerosis and aortic aneurysm ruptures in hypercholesterolemic male mice. J Vasc Surg 70:1658–1668.e1. 30850299 10.1016/j.jvs.2018.11.045 PMC6728232

[B46] Tam K Richards DA Aronovitz MJ Martin GL Pande S Jaffe IZ Blanton RM (2020) Sacubitril/valsartan improves left ventricular function in chronic pressure overload independent of intact cyclic guanosine monophosphate-dependent protein kinase I alpha signaling. J Card Fail 26:769–775. 32464187 10.1016/j.cardfail.2020.04.011 PMC7529795

[B47] Thatcher SE Zhang X Howatt DA Yiannikouris F Gurley SB Ennis T Curci JA Daugherty A Cassis LA (2014) Angiotensin-converting enzyme 2 decreases formation and severity of angiotensin II-induced abdominal aortic aneurysms. Arterioscler Thromb Vasc Biol 34:2617–2623. 25301841 10.1161/ATVBAHA.114.304613 PMC4250973

[B48] Vetrugno L Costa MG Pompei L Chiarandini P Drigo D Bassi F Gonano N Muzzi R Della Rocca G (2012) Prognostic power of pre- and postoperative B-type natriuretic peptide levels in patients undergoing abdominal aortic surgery. J Cardiothorac Vasc Anesth 26:637–642. 22387082 10.1053/j.jvca.2012.01.018

[B49] Vijayaraghavan J Scicli AG Carretero OA Slaughter C Moomaw C Hersh LB (1990) The hydrolysis of endothelins by neutral endopeptidase 24.11 (enkephalinase). J Biol Chem 265:14150–14155. 2201681

[B50] Weckler N Leitzbach D Kalinowski L Malinski T Busch AE Linz W (2003) Effect of chronic treatment with the vasopeptidase inhibitor AVE 7688 and ramipril on endothelial function in atherogenic diet rabbits [published correction appears in *J Renin Angiotensin Aldosterone Syst* (2004) 5:58]. J Renin Angiotensin Aldosterone Syst 4:191–196. 14608526 10.3317/jraas.2003.031

[B51] Wu L Iwai M Nakagami H Li Z Chen R Suzuki J Akishita M de Gasparo M Horiuchi M (2001) Roles of angiotensin II type 2 receptor stimulation associated with selective angiotensin II type 1 receptor blockade with valsartan in the improvement of inflammation-induced vascular injury. Circulation 104:2716–2721. 11723025 10.1161/hc4601.099404

[B52] Yamamoto E Kataoka K Dong YF Nakamura T Fukuda M Tokutomi Y Matsuba S Nako H Nakagata N Kaneko T (2009) Aliskiren enhances the protective effects of valsartan against cardiovascular and renal injury in endothelial nitric oxide synthase-deficient mice. Hypertension 54:633–638. 19597038 10.1161/HYPERTENSIONAHA.109.133884

[B53] Yiannikouris F Karounos M Charnigo R English VL Rateri DL Daugherty A Cassis LA (2012) Adipocyte-specific deficiency of angiotensinogen decreases plasma angiotensinogen concentration and systolic blood pressure in mice. Am J Physiol Regul Integr Comp Physiol 302:R244–R251. 22071160 10.1152/ajpregu.00323.2011 PMC3349391

[B54] Zhang H Liu G Zhou W Zhang W Wang K Zhang J (2019) Neprilysin inhibitor-angiotensin II receptor blocker combination therapy (Sacubitril/valsartan) suppresses atherosclerotic plaque formation and inhibits inflammation in apolipoprotein E- deficient mice. Sci Rep 9:6509. 31019233 10.1038/s41598-019-42994-1 PMC6482143

